# Supplementary material for: Universal Murray’s law for optimised fluid transport in synthetic structures
Source: Nat Commun. 2024 May 7;15:3652. doi: 10.1038/s41467-024-47833-0 (PMC11076523; doi:10.1038/s41467-024-47833-0)
Supplement: Supplementary file 1 — Supplementary Information [file 41467_2024_47833_MOESM1_ESM.pdf]

Supplementary Information

Universal Murray's law for optimised fluid transport in synthetic structures

Binghan Zhou[1], Qian Cheng[2], Zhuo Chen[1], Zesheng Chen[1], Dongfang Liang[2], Eric Anthony Munro[1], Guolin Yun[1], Yoshiki Kawai[3], Jinrui Chen[1], Tynee Bhowmick[1], Padmanathan Karthick Kannan[4], Luigi Giuseppe Occhipinti[1], Hidetoshi Matsumoto[3], Julian William Gardner[4], Bao-Lian Su[5,6] and Tawfique Hasan[1]*

[1]*Cambridge Graphene Centre, University of Cambridge, Cambridge, CB3 0FA, UK*

[2]*Department of Engineering, University of Cambridge, Cambridge, CB2 1PZ, UK*

[3]*Department of Materials Science and Engineering, Tokyo Institute of Technology, Tokyo, 152-8552, Japan*

[4]*School of Engineering, University of Warwick, Coventry, CV4 7AL, UK*

[5]*Laboratory of Inorganic Materials Chemistry (CMI), University of Namur, B-5000 Namur, Belgium*

[6]*State Key Laboratory of Advanced Technology for Materials Synthesis and Processing, Wuhan University of Technology, Wuhan, 430070, China*

**email: th270@cam.ac.uk*

Content

Supplementary Note 1. The initial deduction of Murray’s law. 4

Supplementary Note 2. Deducing Murray’s law by minimising resistance. 6

Supplementary Note 3. The deduction of Universal Murray’s law. 8

Supplementary Table 1. 11

Supplementary Figure 1. 11

Supplementary Note 4. Several derivations of Universal Murray’s law. 12

Supplementary Note 5. Murray’s law in hierarchical tubular network with arbitrary shape. 14

Supplementary Figure 2. 16

Supplementary Figure 3. 17

Supplementary Figure 4. 18

Supplementary Figure 5. 19

Supplementary Figure 6. 19

Supplementary Figure 7. 20

Supplementary Figure 8. 20

Supplementary Figure 9. 21

Supplementary Figure 10. 21

Supplementary Note 6. The construction of optimal planar and tubular structure based on GOA. 22

Supplementary Figure 11. 24

Supplementary Figure 12. 24

Supplementary Figure 13. 25

Supplementary Figure 14. 25

Supplementary Figure 15. 26

Supplementary Figure 16. 26

Supplementary Figure 17. 27

Supplementary Figure 18. 27

Supplementary Figure 19. 28

Supplementary Figure 20. 29

Supplementary Figure 21. 30

Supplementary References.. 31

Supplementary Note 1. The initial deduction of Murray's law. In 1926, Cecil D. Murray deduced the optimal hierarchical structure of branching vessels in animals with minimal energy consumption for blood flow.[1] He assumed the total power consumption of the vascular system P_t as the sum of consumption to overcome the resistance of laminar flow P_f, and the respiratory power to maintain the biological activity of the vessels P_m:

$$P_t = P_f + P_m \qquad (S.1)$$

Neglecting the gravitational potential and kinetic energy terms, Hagen-Poiseuille's law[2] gives the volumetric flow rate Q for incompressible laminar flow in a circular tube as:

$$Q = \frac{\pi r^4}{8\eta l} \Delta p \qquad (S.2)$$

where r is the radius of the tube, η is the fluid viscosity, l is the length of the tube, and Δp is the pressure difference along the tube. Supposing the tube length $l = 1$, and letting constant $a = \frac{8\eta}{\pi}$, the power term of flow resistance can be written as:

$$P_f = Q\Delta p = aQ^2 r^{-4} \qquad (S.3)$$

Murray assumed that the metabolic power required to sustain the regular physiological activity of both the vessel and its content is linear to the volume:

$$P_m = m\pi r^2 l = br^2 \qquad (S.4)$$

where m is the metabolic power per unit volume of the vessel, and constant $b = m\pi$.

Then, the total power needed to maintain the blood flow for a tube of unit length can be written as:

$$P_t = P_f + P_m = aQ^2 r^{-4} + br^2 \qquad (S.5)$$

Q is also regarded as a constant here, assuming the organism requires a fixed blood flow for maintaining physiological activities. Hence, P_t depends only on r, and P_t reaches the minimum when:

$$\frac{dP_t}{dr} = -4aQ^2 r^{-5} + 2br = 0 \qquad (S.6)$$

$$\frac{d^2 P_t}{dr^2} = 20aQ^2 r^{-6} + 2b > 0 \qquad (S.7)$$

The inequality $(S.7)$ is apparent as a, Q, r, b are all positive. The solution of Equ. $(S.6)$ is given by:

$$Q = \sqrt{\frac{b}{2a}} r^3 \tag{S.8}$$

Let constant $\sqrt{\frac{b}{2a}} = k$, $Q = kr^3$. Assuming the total volumetric flow rate Q is conserved in the network, the sum of Q in branching channels equals the inlet flow rate Q_0:

$$Q_0 = \sum Q_t \tag{S.9}$$

where subscript t represents the level number of the tubes ($t = 1, 2, 3, \cdots$). Putting into $Q = kr^3$:

$$\sum r_1^3 = \sum r_2^3 = \cdots = \frac{Q_1}{k} = a\ constant \tag{S.10}$$

The above equation is Murray's law, named after its proposer.

Supplementary Note 2. Deducing Murray's law by minimising resistance. Considering an i-level hierarchical tubular network such as Fig. 1a, where $r_1, r_2, \cdots, r_i$ represent the radii of pores at each level, $n_1, n_2, \cdots, n_i$ are the numbers of pores, and $l_1, l_2, \cdots, l_i$ are the tube lengths. The constraint condition for deduction is the fixed total volume $V = \sum_{t=1}^{i} \pi n_t r_t^2 l_t = V_0$.

For laminar flow, considering Hagen-Poiseuille's law, the laminar flow resistance could be written as[3]:

$$R = \frac{\Delta p}{Q} = \frac{8\eta}{\pi} \cdot \frac{l}{r^4} = k_1 \frac{l}{r^4} \qquad (S.11)$$

where $k_1 = \frac{8\eta}{\pi}$.

Analog to electrical resistance, the resistance of series-connected pipes or pipe systems should be the sum of each component, as the pressure drop is accumulated along the series connection with the same Q. Assuming the total flow current equally distributes into every single tube, the resistance of parallel-connected same pipes should be the resistance of a single pipe over the number of pipes: $R_{parallel-connection} = \frac{R_{single}}{n}$, where $R_{parallel-connection}$ is the total resistance of the parallel-connected system, R_{single} is the resistance of a single pipe, and n represents the number of parallel-connected pipes. If the flow evenly distributes according to the inverse ratios of resistance, the total resistance's inverse is the sum of the inverses of the parallel-connected pipes' resistances. Because the total Q would be the sum of flow currents in every single tube under the same Δp, lowering the total resistance in inverse proportion.

The discussed hierarchical tubular network is series-connected from levels of parallel-connected pipes. Therefore, the total resistance of the network could be written as:

$$R_{total} = \sum_{t=1}^{i} R_t = k_1 \sum_{t=1}^{i} \frac{l_t}{n_t r_t^4} \qquad (S.12)$$

Lagrange multiplier method is utilised to minimise the total resistance R_t by introducing constraint $V - V_0 = 0$ and constructing the optimisation function:

$$\Pi = R_{total} - \lambda(V - V_0) = k_1 \sum_{t=1}^{i} \frac{l_t}{n_t r_t^4} - \lambda \left(\sum_{t=1}^{i} \pi n_t r_t^2 l_t - V_0 \right) \qquad (S.13)$$

where λ is the Lagrange multiplier. To minimise R_t, let the partial derivatives of Π to each variable r equal zero:

$$\frac{\partial \Pi}{\partial r_t} = -\frac{4k_1 l_t}{n_t r_t^5} - 2\pi n_t \lambda l_t r_t = 0, \qquad t = 1,2,\cdots,i \tag{S.14}$$

Solving the functions, we have the cubic form of Murray's law for laminar flow:

$$n_1 r_1^3 = n_2 r_2^3 = \cdots = n_i r_i^3 = \sqrt{-\frac{2k_1}{\pi\lambda}} = a\ constant \tag{S.15}$$

$$\sum r_1^3 = \sum r_2^3 = \cdots = \sum r_i^3 \tag{S.16}$$

Similarly, for diffusion ($R = \frac{\Delta C}{Q}$) and ionic migration ($R = \frac{\Delta V}{Q}$), the minimisation of resistance also gives the square form of Murray's law:

$$n_1 r_1^2 = n_2 r_2^2 = \cdots = n_i r_i^2 \tag{S.17}$$

Supplementary Note 3. The deduction of Universal Murray's law. Considering a hierarchical system of straight and fractal branches with a fixed total volume as the boundary condition. For a proper size variable x grown with the cross-sectional area, such as radius r for a circular tube or side length for a rectangular tube, assuming the cross-section area A can generally be written as:

$$A = k_1 x^{\alpha} \qquad (S.18)$$

where k_1 is the shape constant, and α is the exponent of x. Both k_1 and α are given by the section shape and the selection of x.

Supposing there is a mass flow caused by a generalised potential difference ΔP in the network, and the transfer rate Q is linearly proportional to the pressure difference over the length: $Q \propto \frac{\Delta P}{l}$. Assuming the transfer rate Q varies as a power of x, it can be expressed as:

$$Q = k_2 x^{\beta} \cdot \frac{\Delta P}{l} \qquad (S.19)$$

where k_2 is the linear coefficient, and β is the exponent of x. For example, Hagen-Poiseuille's law gives $Q = \frac{\pi r^4}{8\eta l}\Delta p$ for laminar flow in circular pipe, thus, $x = r$, $k_2 = \frac{\pi}{8\eta l}$, and $\beta = 4$.

Similar to the discussion in laminar flow, the resistance of such a process is $R = \frac{\Delta p}{Q} = \frac{l}{k_2 x^{\beta}}$. Consequently, for an i-level network with the branch lengths of $l_1, l_2, \cdots, l_i$, branch numbers of $n_1, n_2, \cdots, n_i$, and branch sizes of $x_1, x_2, \cdots, x_i$, the total resistance of the network could be written as:

$$R_{total} = \sum_{t=1}^{i} R_t = \frac{1}{k_2}\sum_{t=1}^{i} \frac{l_t}{n_t x_t^{\beta}} \qquad (S.20)$$

Considering the constraint condition of a given total volume $V = \sum_{t=1}^{i} n_t S_t l_t = k_1 \sum_{t=1}^{i} n_t x_t^{\alpha} l_t = V_0$, Lagrange multiplier method gives the optimisation function:

$$\Pi = R_{total} - \lambda(V - V_0) = \frac{1}{k_2}\sum_{t=1}^{i} \frac{l_t}{n_t x_t^{\beta}} - \lambda\left(k_1 \sum_{t=1}^{i} n_t x_t^{\alpha} l_t - V_0\right) \qquad (S.21)$$

To minimise R_t:

$$\frac{\partial \Pi}{\partial x_t} = -\frac{\beta l_t}{k_2 n_t} x_t^{-\beta-1} - \lambda k_1 \alpha n_t l_t x_t^{\alpha-1} = 0, t = 1,2,\cdots,i \qquad (S.22)$$

setting constant $k_3 = -\frac{\beta}{\lambda k_1 k_2 \alpha}$, we have:

$$n_1^2 x_1^{\alpha+\beta} = n_2^2 x_2^{\alpha+\beta} = \cdots = n_i^2 x_i^{\alpha+\beta} = k_3 \qquad (S.23)$$

Then we obtain the Universal Murray's law:

$$n_1 x_1^{(\alpha+\beta)/2} = n_2 x_2^{(\alpha+\beta)/2} = \cdots = n_i x_i^{(\alpha+\beta)/2} \qquad (S.24)$$

$$\sum x_1^{(\alpha+\beta)/2} = \sum x_2^{(\alpha+\beta)/2} = \cdots = \sum x_i^{(\alpha+\beta)/2} \qquad (S.25)$$

Notably, the restrictions made on lengths l_t do not affect the results, such as assuming a proportional relationship between the length and channel size, $l_t \propto x_t$, or the channel length halved at each level, $l_{t+1} = \frac{l_t}{2}$. This is because the lengths l_t are eliminated in Eq. $(S.22)$.

More generally, when the channels' shapes or fractal structure at different levels are inconsistent as shown in Supplementrary Fig. 1, the section area and flow rate expressions would have unequal exponents and constants. At the t-level channel, the above two formulae would be written as: $A_t = k_{1,t} x_t^{\alpha_t}$, and $Q_t = k_{2,t} x_t^{\beta_t} \cdot \frac{\Delta P}{l}$. The following process also gives similar equations as $(S.22)$, $\frac{\partial \Pi}{\partial x_t} = -\frac{\beta_t l_t}{k_{2,t} n_t} x_t^{-\beta_t - 1} - \lambda k_{1,t} \alpha_t n_t l_t x_t^{\alpha_t - 1} = 0$. However, the different $k_{1,t}$, $k_{2,t}$, α_t, and β_t the constant $k_{3,t} = \frac{\beta_1}{k_{1,1}\, k_{2,1}\, \alpha_1}$ cannot be eliminated in the next step. Equation $(S.25)$ in this case transforms into the ratio given below:

$$\sum x_1^{(\alpha_1+\beta_1)/2} : \cdots : \sum x_i^{(\alpha_i+\beta_i)/2} = \sqrt{\frac{\beta_1}{k_{1,1}\, k_{2,1}\, \alpha_1}} : \cdots : \sqrt{\frac{\beta_i}{k_{1,i}\, k_{2,i}\, \alpha_i}} \qquad (S.26)$$

Additionally, a similar deduction can be applied in circumstances where the mass transfer rate Q is not linearly proportional to the pressure difference, but can be expressed as below:

$$Q = k_2 x^{\beta} \cdot \left(\frac{\Delta P}{l}\right)^{\gamma}, \qquad \gamma \neq 1 \qquad (S.27)$$

The resistance of such a process becomes $R = \frac{\Delta p}{Q} = \frac{1}{k_2} x^{-\beta} \Delta P^{1-\gamma} l^{\gamma}$. Notably, the resistance of parallel-connected identical pipes would also be $R_{parallel-connection} = R_{single} \cdot n^{-1}$, where $R_{parallel-connection}$ is the total resistance of the parallel-connected system, R_{single} is the resistance of a single pipe, and n represents the number of parallel-connected pipes. This apparent relationship can easily be obtained by carefully analysing the evenly distributed transfer rate Q/n and ΔP between the two sides of the pipes. Thus, in the discussed hierarchical structure, the total resistance should be:

$$R_{total} = \sum_{t=1}^{i} R_t = \frac{1}{k_2} \sum_{t=1}^{i} n_t^{-1} x_t^{-\beta} \Delta P_t^{1-\gamma} l_t^{\gamma} \qquad (S.28)$$

The optimisation function would be:

$$\Pi = \frac{1}{k_2} \sum_{t=1}^{i} n_t^{-1} x_t^{-\beta} \Delta P_t^{1-\gamma} l_t^{\gamma} - \lambda \left(k_1 \sum_{t=1}^{i} n_t x_t^{\alpha} l_t - V_0 \right) \qquad (S.29)$$

$$\frac{\partial \Pi}{\partial x_t} = -k_2^{-1} \beta n_t^{-1} x_t^{-\beta-1} \Delta P_t^{1-\gamma} l_t^{\gamma} - \lambda k_1 \alpha n_t l_t x_t^{\alpha-1} = 0, \qquad t = 1,2,\cdots,i \qquad (S.30)$$

Let constant $k_3 = -\frac{\beta}{\lambda k_1 k_2 \alpha}$, the above equation reduces to:

$$\left(\frac{\Delta P_t}{l_t} \right)^{1-\gamma} = k_3 n_t^2 x_t^{\alpha+\beta}, \qquad t = 1,2,\cdots,i \qquad (S.31)$$

$$\left(\frac{\Delta P_t}{l_t} \right)^{\gamma} = k_3^{\frac{\gamma}{1-\gamma}} n_t^{\frac{2\gamma}{1-\gamma}} x_t^{\frac{\gamma(\alpha+\beta)}{1-\gamma}}, \qquad t = 1,2,\cdots,i \qquad (S.32)$$

Putting $(S.32)$ back into the expression $Q = k_2 x^{\beta} \cdot \left(\frac{\Delta P}{l} \right)^{\gamma}$, and setting constant $k_4 = k_2 k_3^{\frac{\gamma}{1-\gamma}}$, we have the transfer rate in the single pipe at each level:

$$Q_t = k_4 n_t^{\frac{2\gamma}{1-\gamma}} x_t^{\frac{\gamma\alpha+\beta}{1-\gamma}}, \qquad t = 1,2,\cdots,i \qquad (S.33)$$

Then, considering the conservation of total mass transfer: $n_1 Q_1 = n_2 Q_2 = \cdots = n_i Q_i$, we get the expression of Universal Murray's law for Q that is non-linear to ΔP:

$$n_1 x_1^{\frac{\gamma\alpha+\beta}{1+\gamma}} = n_2 x_2^{\frac{\gamma\alpha+\beta}{1+\gamma}} = \cdots = n_i x_i^{\frac{\gamma\alpha+\beta}{1+\gamma}} \qquad (S.34)$$

Although we assume $\gamma \neq 1$ in the above deduction, if substituting $\gamma = 1$, Equ. $(S.34)$ will still degrade into $n_1 x_1^{(\alpha+\beta)/2} = \cdots = n_i x_i^{(\alpha+\beta)/2}$.

Supplementary Table 1. Expressions of Murray's law in hierarchical circular tube networks where $\alpha = 2$.

Mass transfer type	Transfer rate	β	$(\alpha+\beta)/2$	Murray's law expression
Laminar flow	$Q = \frac{\pi}{8\eta} \cdot r^4 \cdot \frac{\Delta p}{l}$	4	3	$\sum r_1^3 = \sum r_2^3 = \cdots = \sum r_i^3$
Diffusion	$Q = D\pi \cdot r^2 \cdot \frac{\Delta C}{l}$	2	2	$\sum r_1^2 = \sum r_2^2 = \cdots = \sum r_i^2$
Ionic migration or electron transportation	$Q = \sigma\pi \cdot r^2 \cdot \frac{\Delta V}{l}$	2	2	$\sum r_1^2 = \sum r_2^2 = \cdots = \sum r_i^2$
Knudsen Diffusion	$Q \propto r^3 \cdot \frac{\Delta C}{l}$	3	2.5	$\sum r_1^{2.5} = \sum r_2^{2.5} = \cdots = \sum r_i^{2.5}$

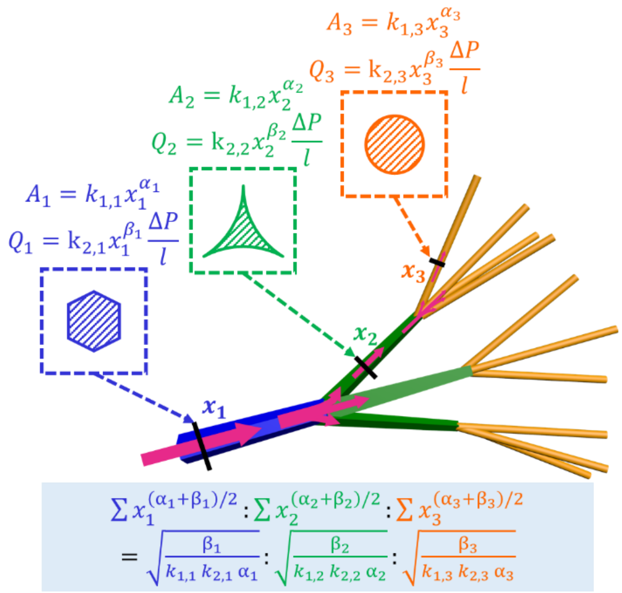

Supplementary Figure 1. Schematic illustration of hierarchical structure with inconstant pore shapes at different levels and the corresponding expression of Universal Murray's law for structure optimisation.

Supplementary Note 4. Several derivations of Universal Murray's law. The following analysis demonstrates how Equ. $(S.34)$ of Universal Murray's law can be used to optimise a few complex situations.

For turbulent flow in a rough tube described by the Darcy-Weisbach equation[3]:

$$\frac{\Delta p}{l} = f_D \frac{\rho}{2} \frac{\langle v \rangle^2}{D_H} \qquad (S.35)$$

where Δp is the pressure drop, l is the tube length, f_D represents the Darcy friction factor, ρ is the density of the fluid, $\langle v \rangle$ is the mean flow velocity, and D_H is the hydraulic diameter. For a circular tube, $D_H = 2r$, where r is the radius. Therefore, for such turbulent flow in rough circular pipes, the volumetric flow rate Q can be written as:

$$Q = \langle v \rangle \cdot A = k_2 r^{\frac{5}{2}} \left(\frac{\Delta p}{l}\right)^{\frac{1}{2}} \qquad (S.36)$$

where $A = \pi r^2$ is the section area, and the constant $k_2 = \frac{2\pi}{\sqrt{f_D \rho}}$. According to Universal Murray's law, $\alpha = 2$ for circular tubes, and the exponents in the above equation give $\beta = \frac{5}{2}$ and $\gamma = \frac{1}{2}$. Consequently, the expression of the optimal hierarchical network for turbulent flow should be:

$$n_1 r_1^{\frac{7}{3}} = \cdots = n_i r_i^{\frac{7}{3}} \qquad (S.37)$$

Turbulent flow in a smooth tube also follows Equ. $(S.35)$. However, an empirical equation with the diameter and flow rate is commonly used for the Darcy friction factor[4]:

$$f_D = 0.046\left(\frac{2Q}{\mu \pi r}\right)^{-\frac{1}{5}} \qquad (S.38)$$

We notice that in the expression of volumetric flow rate Equ. $(S.36)$, the constant $k_5 \propto f_D^{-\frac{1}{2}}$. Therefore, putting $(S.38)$ into $(S.36)$, the flow rate for turbulent flow in a smooth pipe can be expressed as:

$$Q^{\frac{9}{10}} \propto r^{\frac{24}{10}} \left(\frac{\Delta p}{l}\right)^{\frac{1}{2}} \qquad (S.39)$$

$$Q \propto r^{\frac{24}{9}} \left(\frac{\Delta p}{l}\right)^{\frac{5}{9}} \qquad (S.40)$$

According to Universal Murray's law, $\alpha = 2$ for circular tubes, and Equ. $(S.40)$ gives $\beta = \frac{24}{9}$ and $\gamma = \frac{5}{9}$. Thus, based on Equ. $(S.34)$, the optimisation equation for turbulent flow in a smooth tube can be written as:

$$n_1 r_1^{\frac{17}{7}} = \cdots = n_i r_i^{\frac{17}{7}} \qquad (S.41)$$

Due to different empirical assumptions, the very little difference in exponents for turbulent flow in rough and smooth tubes leads to different optimisation expressions, Equ. (S.37) and (S.41) respectively.

For non-Newtonian power-law liquid, the shear stress τ follows $\tau = k(\frac{\partial u}{\partial y})^n$, where k is the flow consistency index, $\frac{\partial u}{\partial y}$ is the velocity gradient perpendicular to the plane of shear, and n denotes the flow behaviour index. The volumetric flow rate of power-law liquid in the circular tube can be written as[5]:

$$Q = (\frac{n\pi}{3n+1})(\frac{\Delta P}{2kl})^{1/n} r^{(3n+1)/n} = k_2 r^{(3n+1)/n} \left(\frac{\Delta p}{l}\right)^{1/n} \qquad (S.42)$$

where r is the radius of the tube and $k_2 = \frac{n\pi}{3n+1} \cdot (2k)^{-1/n}$. For circular tube $\alpha = 2$, and the exponents in $(S.42)$ give $\beta = \frac{3n+1}{n}$ and $\gamma = \frac{1}{n}$. In this circumstance, the exponent in Equ. $(S.34)$ has $\frac{\gamma\alpha+\beta}{1+\gamma} = 3$. The cubic expression of Murray's law is still valid:

$$n_1 r_1^3 = n_2 r_2^3 = \cdots = n_i r_i^3 \qquad (S.43)$$

The above optimisation formula $(S.37)$, $(S.41)$, and $(S.43)$ match the reported results for turbulent flow in rough[6] and smooth[7] pipes, and for the laminar flow of power-law liquid[5], respectively.

Supplementary Note 5. Murray's law in hierarchical tubular network with arbitrary shape. Considering the laminar flow in a tubular branching network with an unidentified closed geometric shape, as shown in Fig. 1c, the hydraulic diameter of the tube is:

$$D_H = \frac{4A}{P} \qquad (S.44)$$

where A is the cross-sectional area and P represents the perimeter. Therefore, the cross-sectional area can be expressed as:

$$A = k_1 D_H^2 \qquad (S.45)$$

where constant $k_1 = \frac{P}{4D_H} = \frac{P^2}{16A}$ is only related to the channel shape. The Reynolds number of the tube shall be:

$$R_e = \frac{D_H v \rho}{\eta} \qquad (S.46)$$

where v is flow speed, ρ is the density of the fluid, and η is the dynamic viscosity of the fluid. According to the Darcy-Weisbach's Formula[8], the friction loss is:

$$h_f = \lambda \frac{l}{D_H} \frac{v^2}{2g} \qquad (S.47)$$

where λ is the resistance coefficient. The dimensional analysis gives that the resistance coefficient should only be a function of the Reynolds number with smooth wall assumption.[9] Note that Darcy-Weisbach's Formula is applicable to all flow regimes. Here, we apply it to the analysis of the laminar case.

In a laminar flow, since the pressure drop along the flow overcomes the viscous shear force at the pipe wall, it is linearly proportional to the flow velocity. Bearing these two considerations, the resistance coefficient becomes inversely proportional to the Reynolds number:

$$\lambda = \frac{k}{R_e} \qquad (S.48)$$

where k is a linear coefficient, specifically for a circular tube $k = 64$. Thus, the pressure difference between the tube shall be:

$$\Delta p = h_f \rho g = \frac{\lambda l\, v^2 \rho}{2D_H} = \frac{k l \eta v}{2D_H^2} \qquad (S.49)$$

Therefore, the volumetric flow rate can be written as:

$$Q = vA = \frac{2k_1}{k\eta} \cdot D_H^4 \cdot \frac{\Delta p}{l} = k_2 \cdot D_H^4 \cdot \frac{\Delta p}{l} \qquad (S.50)$$

where the constant $k_2 = \frac{2k_1}{k\eta}$. In a circular tube, the above equation shows as Hagen-Poiseuille's law.

Putting the above expression of Q and the expression of section area $A = k_1 D_H^2$ into Universal Murray's law, we get the following optimisation of the network for laminar flow:

$$\sum D_{H,1}^3 = \sum D_{H,2}^3 = \cdots = \sum D_{H,i}^3 \qquad (S.51)$$

Common length variables, such as side length, diameter, or perimeter, are proportional to the hydraulic diameter D_H. Thus, for a common length variable x, $A \propto x^2$, $Q \propto x^4$, and Murray's law in tubular network with arbitrary shape can also be written as the cubic form:

$$\sum x_1^3 = \sum x_2^3 = \cdots = \sum x_i^3 \qquad (S.52)$$

The proof for diffusion and ionic migration or electron transportation is straightforward. For optimising the ionic or electronic transfer in such a hierarchical tubular network, Pouillet's law and Ohm's law give the transfer rate as:

$$Q = \frac{\sigma A \Delta V}{l} \qquad (S.53)$$

where σ is the conductivity, A represents the section area, ΔV is the potential difference, and l is the channel length. As for diffusion through the tube, Fick's law gives:

$$Q = \frac{D A \Delta C}{l} \qquad (S.54)$$

where D is the diffusion coefficient, and ΔC is the concentration difference.

Additionally, in a closed 2D geometry (or geometries with infinite depth), the section area is proportional to the square of a typical length variable x: $A \propto x^2$and the transfer rate $Q \propto x^2$. Therefore, according to Universal Murray's law:

$$\sum x_1^2 = \sum x_2^2 = \cdots = \sum x_i^2 \qquad (S.55)$$

Supplementary Table 2. Expressions of Murray's law in the hierarchical planar structure where $\alpha = 1$.

Mass transfer type	Transfer rate	β	$(\alpha + \beta)/2$	Murray's law expression
Laminar flow	$Q = \frac{d}{12\eta} \cdot h^3 \cdot \frac{\Delta p}{l}$	3	2	$\sum h_1^2 = \sum h_2^2 = \cdots = \sum h_i^2$
Diffusion	$Q = Dd \cdot h \cdot \frac{\Delta C}{l}$	1	1	$\sum h_1 = \sum h_2 \cdots = \sum h_i$
Ionic or electronic transfer	$Q = \sigma d \cdot h \cdot \frac{\Delta V}{l}$	1	1	$\sum h_1 = \sum h_2 \cdots = \sum h_i$

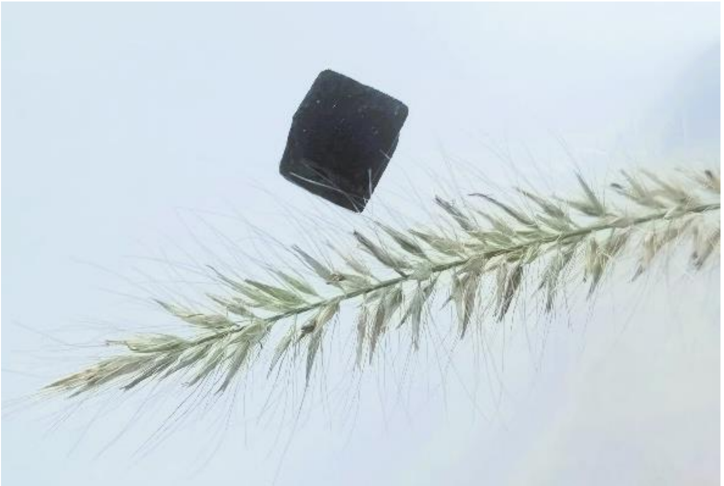

Supplementary Figure 2. GOA on the bristles of green foxtail.

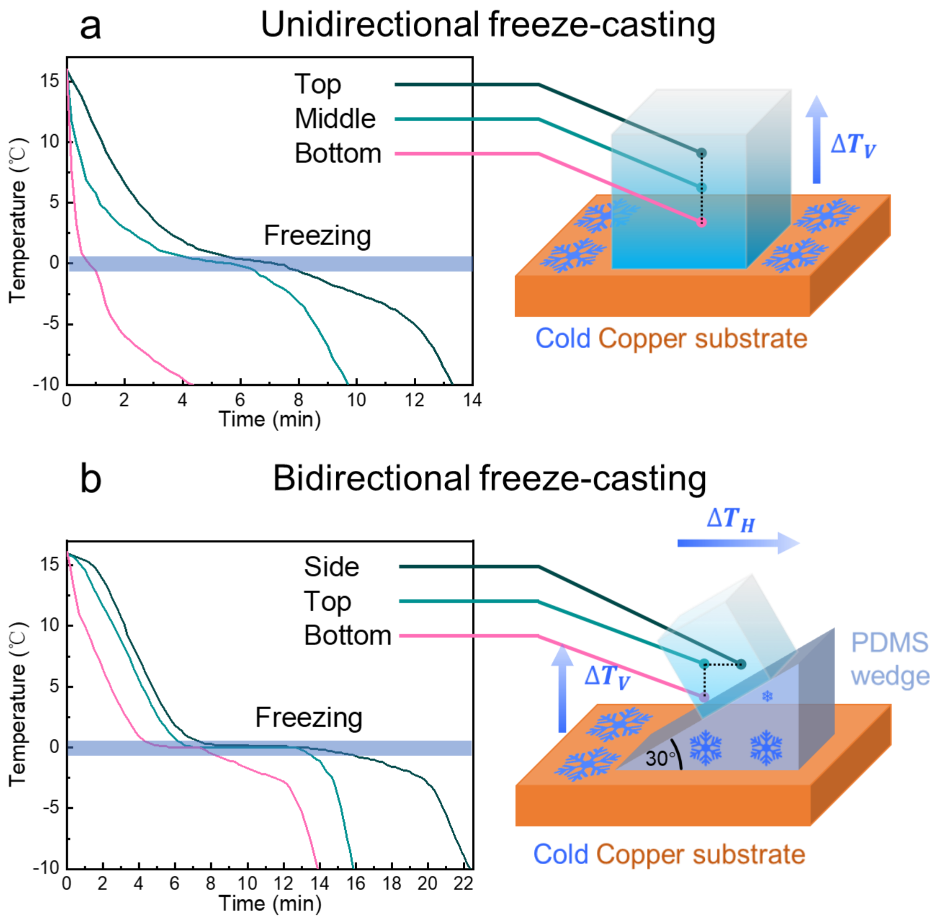

Supplementary Figure 3. Temperature gradient in directional freezing. (a) Temperature changes at different heights during the unidirectional freezing of GO ink at −20 °C. The thermometer probe is inserted at vertical axis along the centre of a 1 cm cube, at the height of 2.5 mm (Bottom), 5 mm (Middle), and 7.5 mm (Top). (b) Temperature changes during the bidirectional freezing of GO ink at −20 °C. The thermometer probe is inserted into a 1 cm cube. The Top and Side are at the same height, and the Top and Bottom are at the same horizontal position.

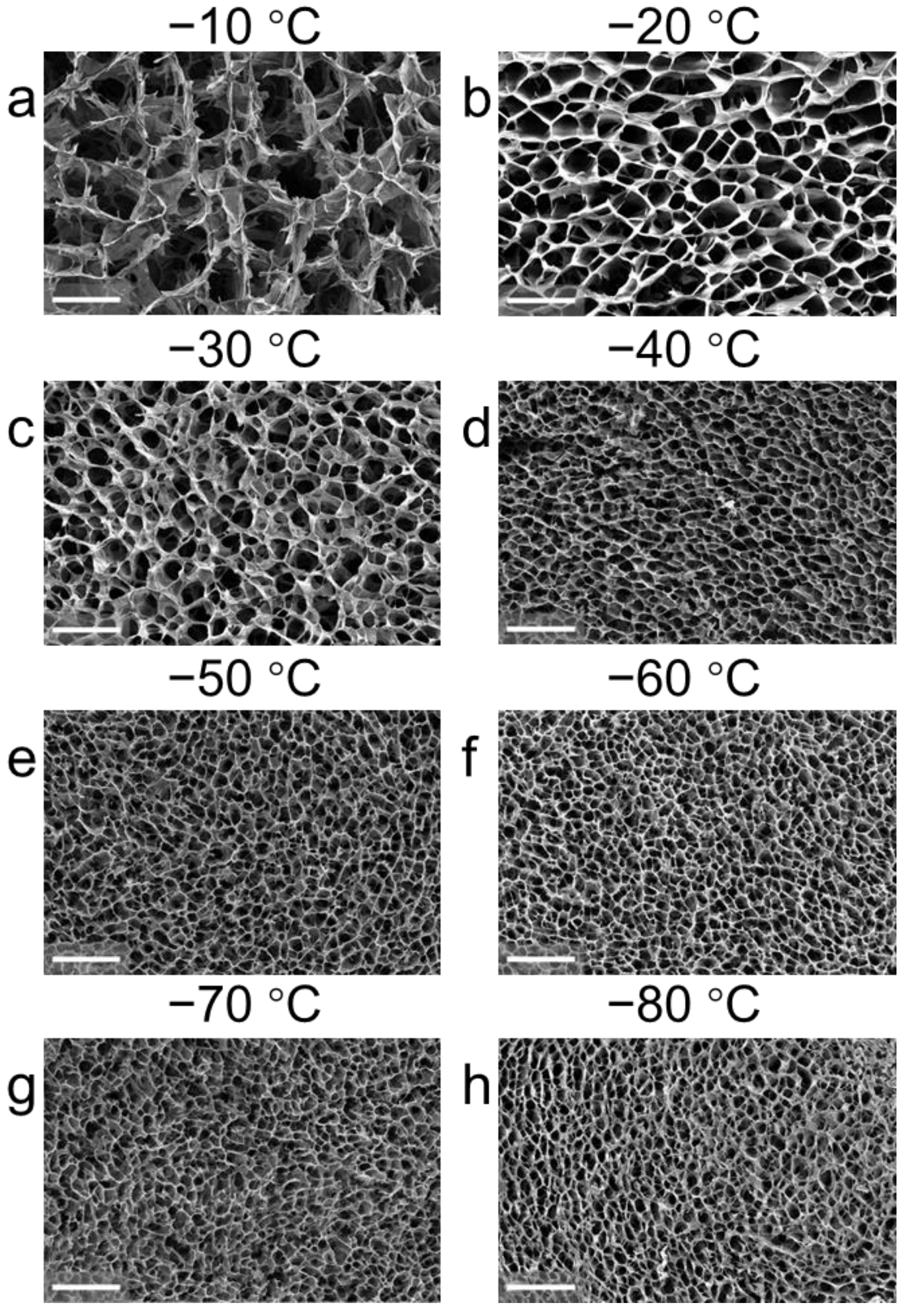

Supplementary Figure 4. Top-view SEM images of unidirectionally freeze-casted GOA frozen at (a) −10 °C, (b) −20 °C, (c) −30 °C, (d) −40 °C, (e) −50 °C, (f) −60 °C, (g) −70 °C, and (h) −80 °C. Scale bars: 100 μm.

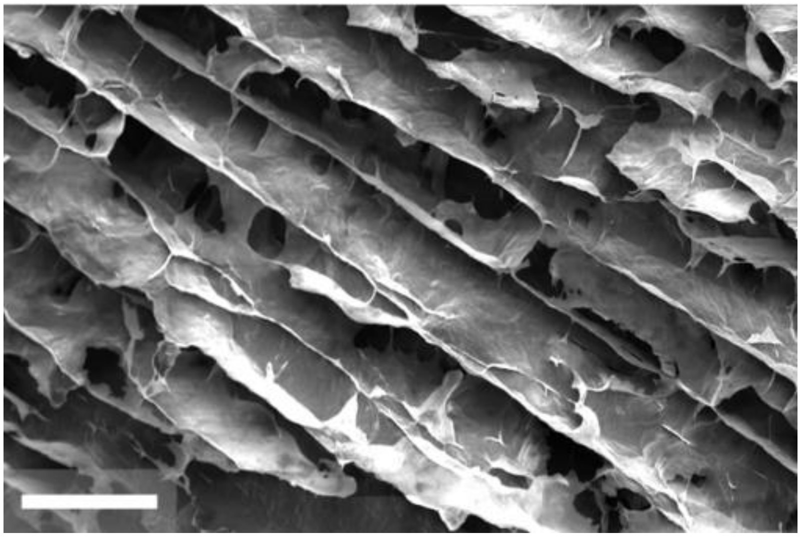

Supplementary Figure 5. Side-view SEM image of the vertically-porous GOA unidirectionally frozen at −20 °C. Scale bars 100 μm.

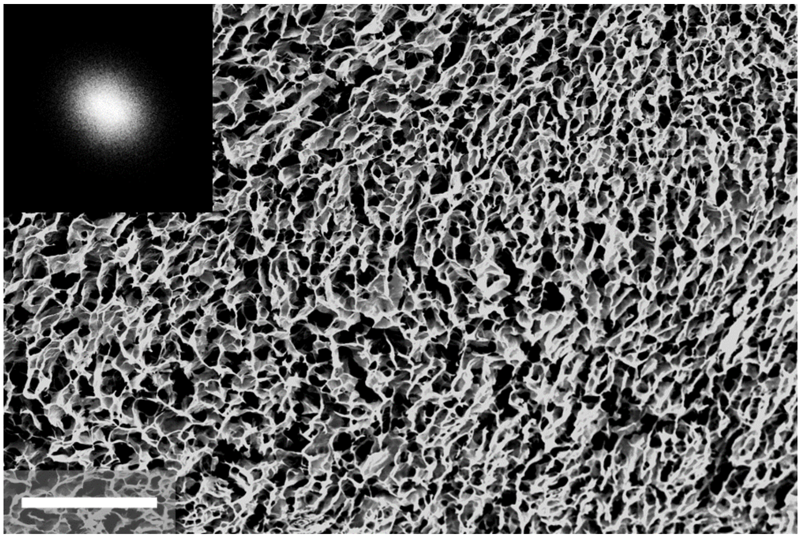

Supplementary Figure 6. Top-view SEM image of GOA frozen by liquid nitrogen at −196 °C. Inset: Fourier transform image. Scale bar: 100 μm.

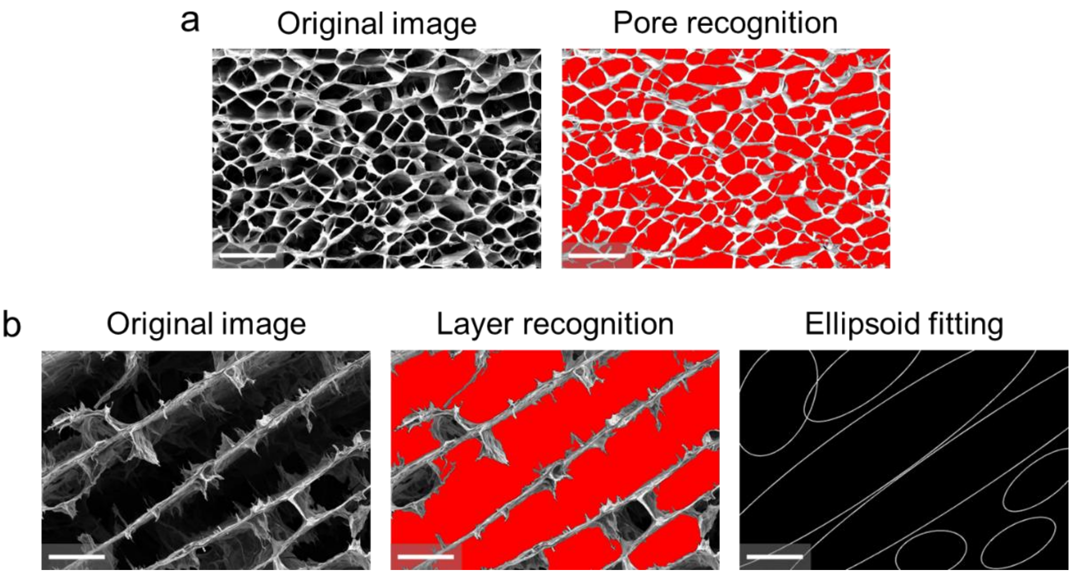

Supplementary Figure 7. (a) Typical pore size measurement process for vertically porous GOA. (b) Typical layer spacing measurement process for lamellar GOA. Scale bars: 100 μm.

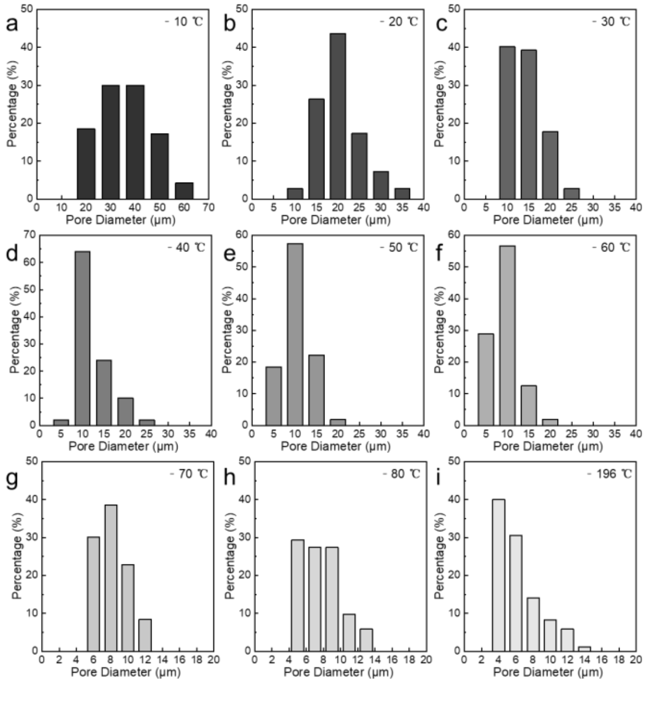

Supplementary Figure 8. Pore diameter distributions of graphene oxide aerogel unidirectionally frozen at (a) −10 ℃, (b) −20 ℃, (c) −30 ℃, (d) −40 ℃, (e) −50 ℃, (f) −60 ℃, (g) −70 ℃, (h) −20 ℃, and (i) frozen in liquid nitrogen at −196 ℃.

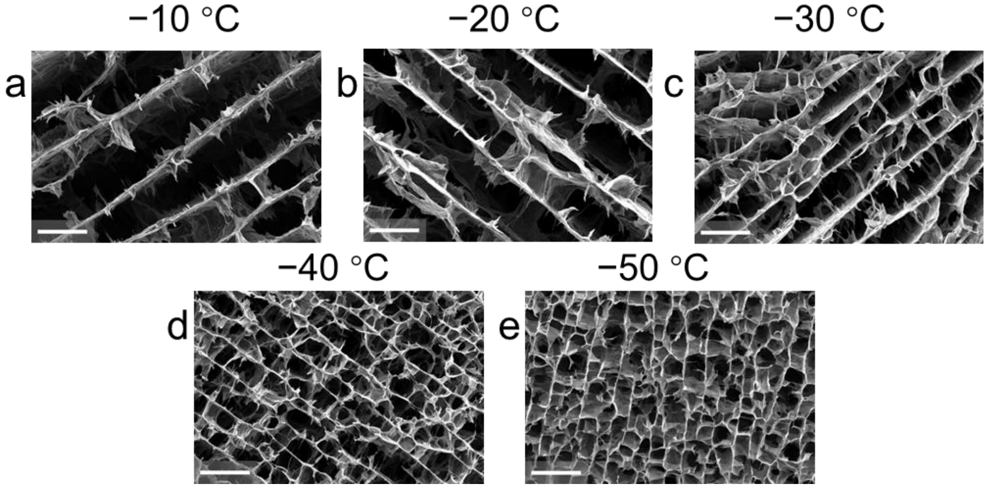

Supplementary Figure 9. Top-view SEM image of bidirectionally freeze-casted GOA frozen at (a) −10 ℃, (b) −20 ℃, (c) −30 ℃, (d) −40 ℃, and (e) −50 ℃. Scale bars: 100 μm.

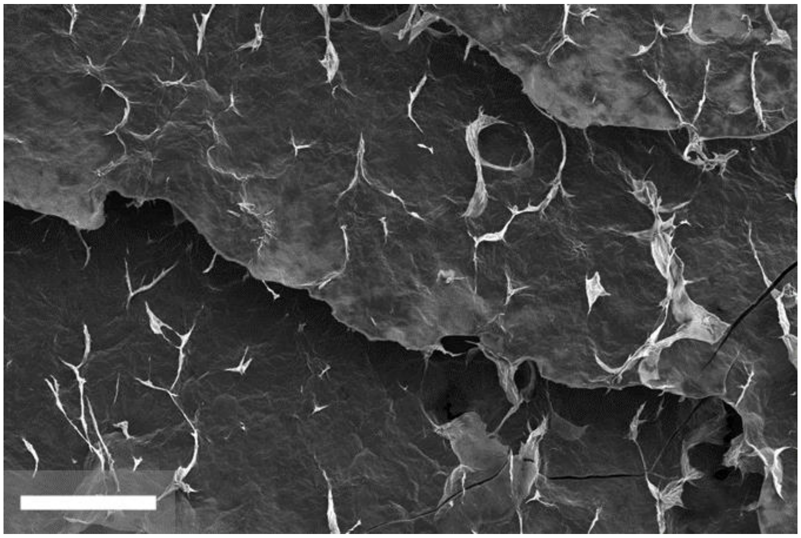

Supplementary Figure 10. Side-view SEM image of lamellar GOA bidirectionally frozen at −10 ℃. Scale bars 100 μm.

Supplementary Note 6. The construction of optimal planar and tubular structure based on GOA. As shown in Supplementary Fig. 11a, we suppose a 3-level hierarchical planar structure with a uniform layer width d. The layer spacings of channels at each level are h_1, h_2, and h_3. The heights of the structure are H_1, H_2, and H_3, respectively. Obviously, the number of channels, n, equals the height over layer spacing:

$$n = \frac{H}{h} \tag{S.56}$$

Universal Murray's law gives the optimisation formula for laminar flow in planar structure, as shown in Supplementary Table 2:

$$n_1 h_1^2 = n_2 h_2^2 = n_3 h_3^2 \tag{S.57}$$

Therefore, we have:

$$H_1 h_1 = H_2 h_2 = H_3 h_3 \tag{S.58}$$

For the planar Murray materials prepared by lamellar GOA bidirectionally freeze-cast at -10 ℃, -30 ℃, -50 ℃, the corresponding average layer spacings at each rank are $h_1 = 97.1\,\mu m$, $h_2 = 50.9\,\mu m$, and $h_3 = 30.5\,\mu m$. Thus, the ratio of the heights is $H_1 : H_2 : H_3 = 0.524 : 1 : 1.67$. The optimal hierarchical structure should be constructed following this ratio to maximise transfer efficiency.

In other hierarchical structures obeying conservation $\sum h_1^1 = \sum h_2^1 = \sum h_3^1$, $\sum h_1^{1.5} = \sum h_2^{1.5} = \sum h_3^{1.5}$, $\sum h_1^{2.5} = \sum h_2^{2.5} = \sum h_3^{2.5}$, $\sum h_1^3 = \sum h_2^3 = \sum h_3^3$, the average channel height can also be calculated through the above process.

Optimising the tubular structure based on vertically porous GOA can be conducted similarly. As shown in Supplementary Fig. 16a, supposing a 3-level pipe with the diameters of D_1, D_2, and D_3, filled with GOA of decreasing pore size r_1, r_2, and r_3, the number of tubular channels at each level, n, can be written as the pipe section area over the section area of a single pore, and thus linear to the square of the pipe diameter over the square of pore size:

$$n = \frac{S_{pipe}}{S_{pore}} \propto \frac{D^2}{r^2} \tag{S.59}$$

Notably, close-packed hexagonal tubes rather than circular pipes are more suitable for describing the vertically porous GOA (Fig. 2a-d). Although the original Murray's law did

not consider this hexagonal model, the previous discussion based on Universal Murray's law about non-circular pipe still allows the optimisation formula for laminar flow:

$$n_1 r_1^3 = n_2 r_2^3 = n_3 r_3^3 \qquad (S.60)$$

Hence:

$$D_1^2 r_1 = D_2^2 r_2 = D_3^2 r_3 \qquad (S.61)$$

For tubular GOA bidirectionally freeze-cast at -20 ℃, -40 ℃, -70 ℃, the corresponding pore radii are $r_1 = 20.6\ \mu m$, $r_2 = 12.6\ \mu m$, and $r_3 = 8.17\ \mu m$. Thus, the optimal ratio of pipe diameters is $D_1 : D_2 : D_3 = 0.782 : 1 : 1.24$. The tubular Murray structure based on vertically porous GOA is designed in accordance with this result. Additionally, the pipe shapes of other tubular hierarchical structures following $\sum r_1^1 = \sum r_2^1 = \sum r_3^1$, $\sum r_1^2 = \sum r_2^2 = \sum r_3^2$, $\sum r_1^4 = \sum r_2^4 = \sum r_3^4$, and $\sum r_1^5 = \sum r_2^5 = \sum r_3^5$ are designed though the same calculation processes.

In the optimisation of the planar and tubular structures, even if not employing Murray's law, the same expressions, $H_1 h_1 = H_2 h_2 = H_3 h_3$ and $D_1^2 r_1 = D_2^2 r_2 = D_3^2 r_3$, can be obtained by directly optimising the resistance on the given structural conditions and constraints.

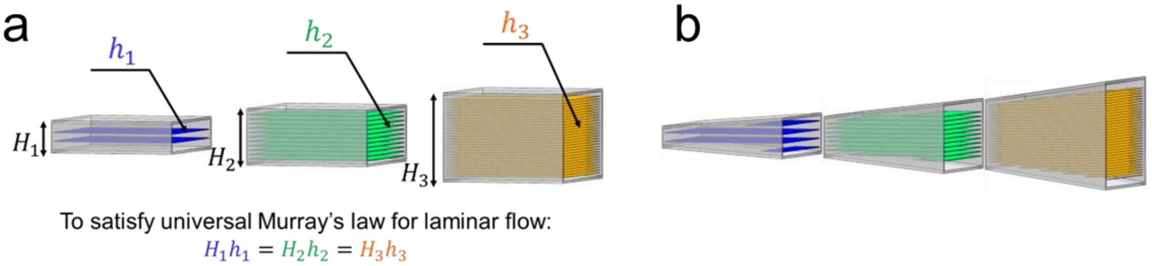

Supplementary Figure 11. Construction of hierarchical planar structure obeying Universal Murray's law. (a) Schematic illustration of ideal optimal planar structure based on lamellar GOA with decreasing layer spacing. Only the central part of the second and third pipes would carry the flow for a simple straight pipe designed by Murray's law with perpendicular connections between sections. The excess concentration of the flow current at the pipe centre contradicts the even-flow assumption in Murray's law and causes huge flow resistance. (b) Schematic illustration of the actual sloping planar structure. The expanding structure could disperse the flow more evenly in the channels, in accordance with the even-flow assumption in the deduction of Murray's law. Since all channels remain parallel except those closest to the inner wall, the deviation of this structure from Murray's law is negligible.

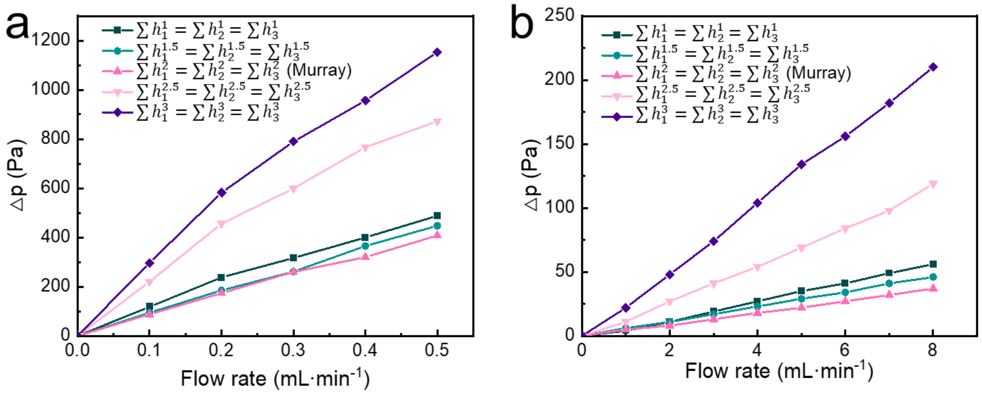

Supplementary Figure 12. The pressure difference for (a) water flow and (b) air flow in hierarchical planar pipes with a fixed total volume.

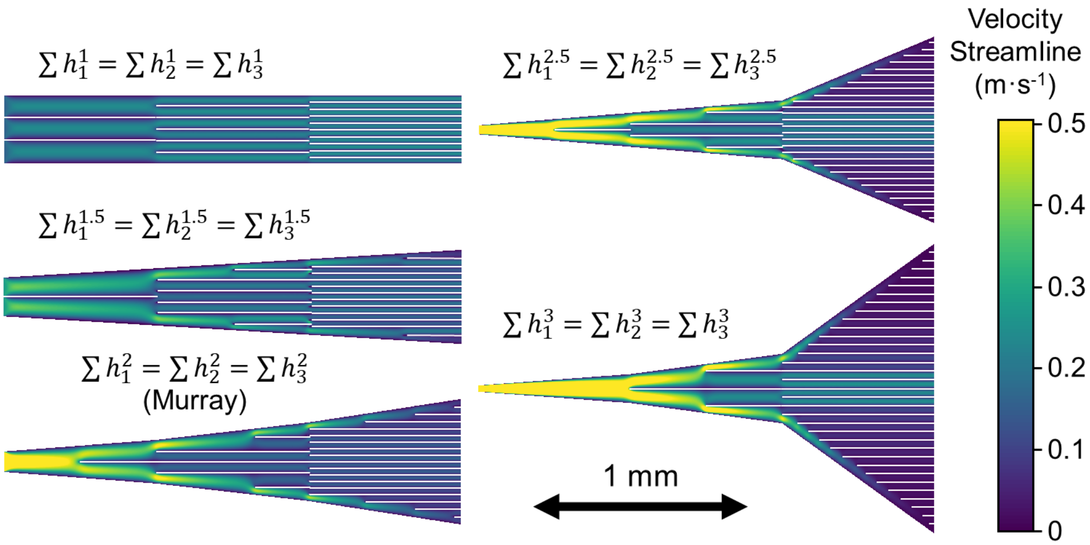

Supplementary Figure 13. Air flow simulation of hierarchical planar structure. The white lines in models represent lateral partitions conform to lamellar GOA layers with corresponding spacing.

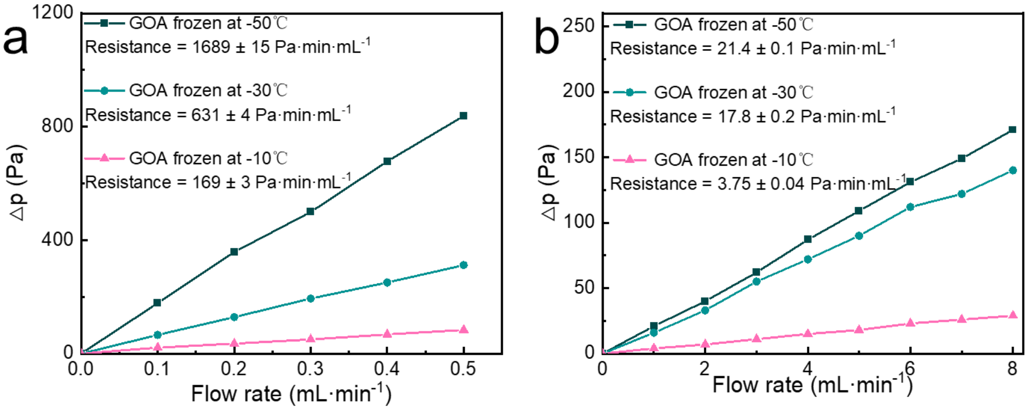

Supplementary Figure 14. The curves of pressure difference versus (a) water and (b) air flow rate and corresponding resistance of lamellar GOA frozen at different temperatures.

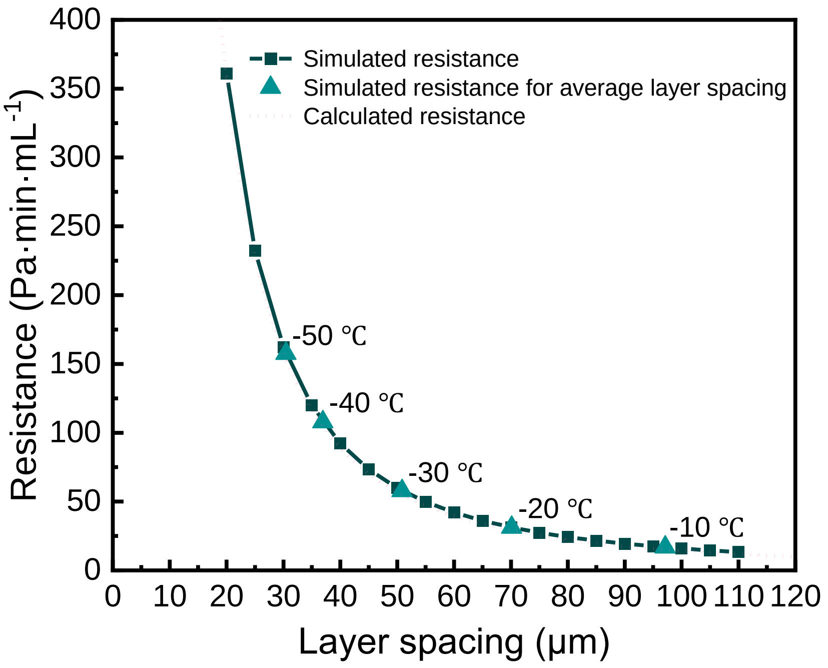

Supplementary Figure 15. Simulated and calculated flow resistance of lamellar structure in straight pipe. The blue triangles refer to the average layer spacing of lamellar GOA bidirectionally freeze-cast at different temperatures. The calculated resistance curve is obtained by $R = \frac{\Delta p}{Q} = \frac{12\eta l}{d} \cdot \frac{1}{nh^3} = \frac{12\eta l}{Hd} \cdot \frac{1}{h^2}$.

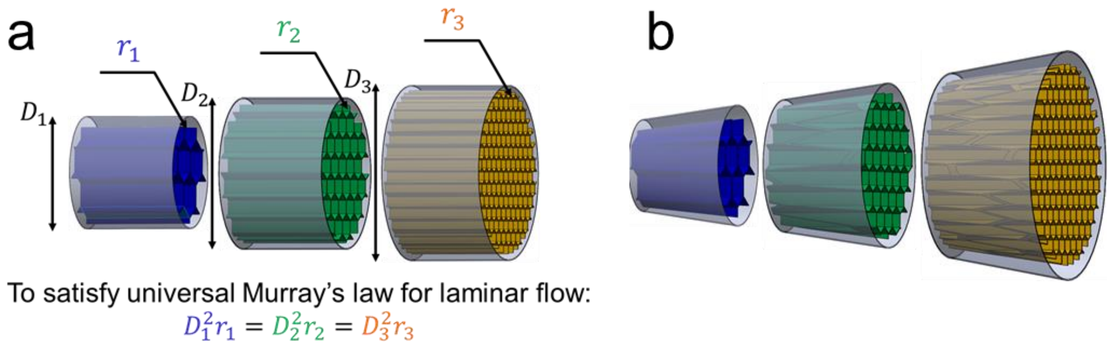

Supplementary Figure 16. Construction of the hierarchical tubular structure obeying Universal Murray's law. Schematic illustration of (a) ideal and (b) actual optimal tubular structure based on vertically porous GOA with shrinking pores.

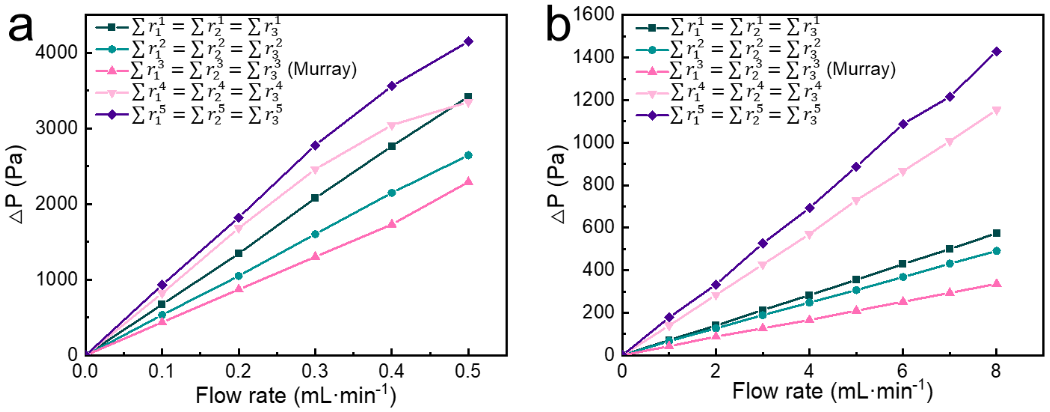

Supplementary Figure 17. The pressure difference for (a) water flow and (b) air flow in hierarchical tubular GOA with a fixed total volume.

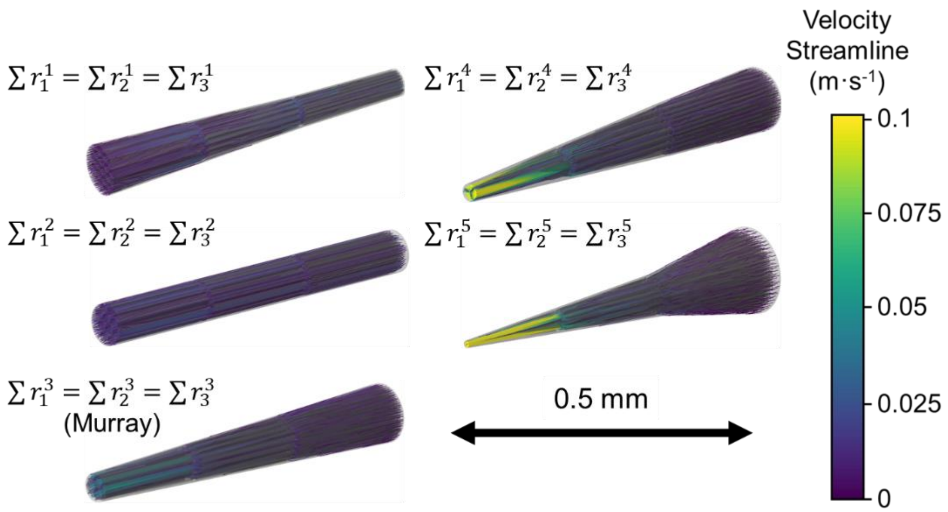

Supplementary Figure 18. Air flow simulation of scaled-down hierarchical tubular structures.

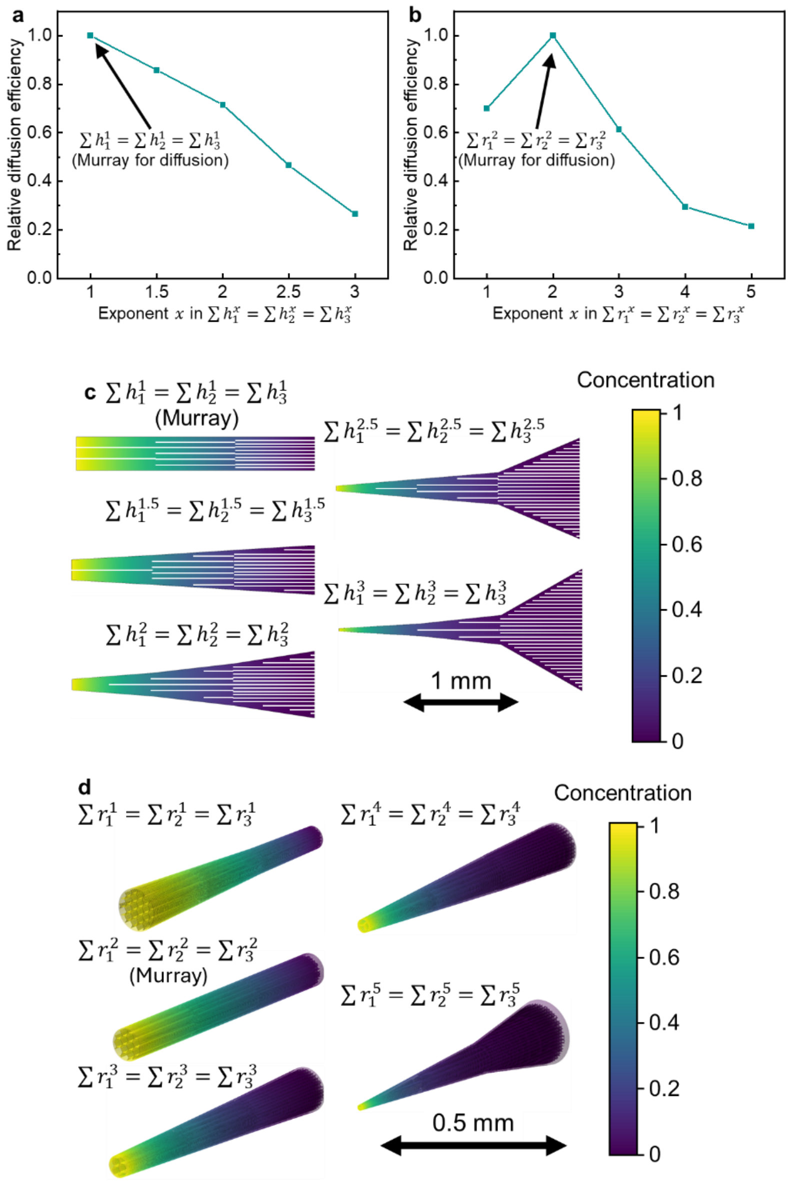

Supplementary Figure 19. Dimensionless diffusion simulation of hierarchical structures. (a) Simulated relative diffusion efficiency of scaled-down hierarchical planar structures. (b) Simulated relative diffusion efficiency of scaled-down hierarchical tubular structures. (c) Dimensionless diffusion simulation of scaled-down hierarchical planar structures. (d) Dimensionless diffusion simulation of scaled-down hierarchical tubular structures. The dimensionless Fickian diffusion is simulated with an inlet concentration (arbitrary unit) of 1 and an outlet of 0. A unity diffusion coefficient is applied. The solutions are seen as converged when the residuals reach 10^{-8}. Using the same boundary conditions, different structures' diffusion fluxes are computed and compared with those following Murray's law as relative diffusion efficiency.

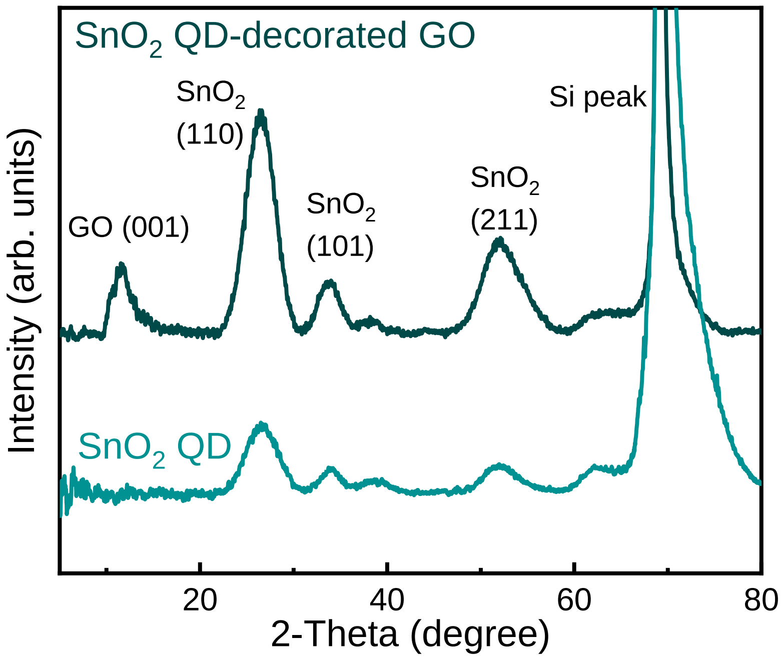

Supplementary Figure 20. XRD patterns of SnO_2 QD-decorated GO and SnO_2 QDs.

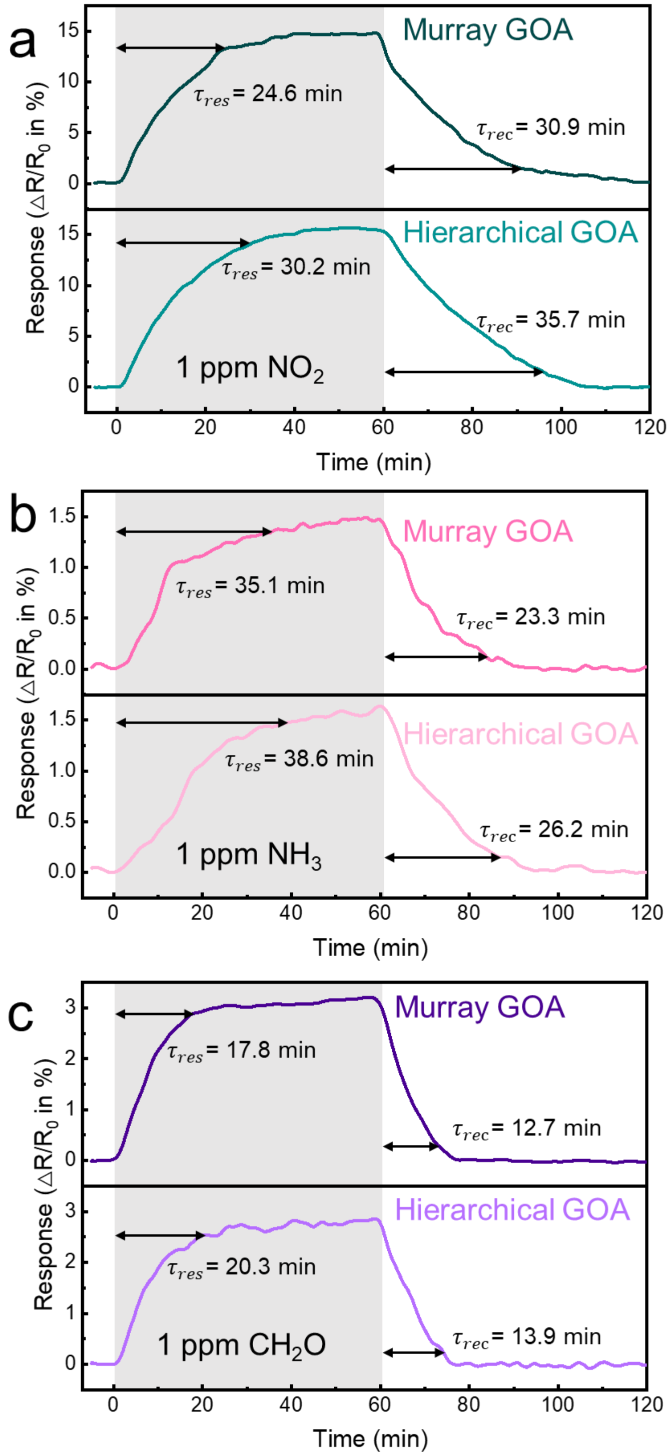

Supplementary Figure 21. Response curves of hierarchical SnO_2 QD-doped GOA in the straight pipe and optimised by Murray's law towards (a) 1 ppm nitrogen dioxide, (b) 1 ppm ammonia, and (c) 1 ppm formaldehyde.

Supplementary References

1 Murray, C. D. The physiologtcal principle of minimom work I The vascular system and the cost of blood volume. *Proc. Natl. Acad. Sci. U. S. A.* **12**, 207-214 (1926).

2 Sutera, S. P. & Skalak, R. The history of Poiseuille's law. *Annu. Rev. Fluid Mech.* **25**, 1-20 (1993).

3 Choi, S., Lee, M. G. & Park, J. K. Microfluidic parallel circuit for measurement of hydraulic resistance. *Biomicrofluidics* **4**, 034110 (2011).

4 Gosselin, L. & Bejan A. Tree networks for minimal pumping power. *Int. J. Therm. Sci.* **44**, 53-63 (2005).

5 Emerson, D. R. & Barber, R. W. A design approach for non-Newtonian power-law flow in rectangular micro-channels based on Murray's law. *Proceedings of the 3rd European Conference on Microfluidics.* μFLU12-235 (2012).

6 Stephenson, D. & Lockerby, D. A. A generalized optimization principle for asymmetric branching in fluidic networks. *Proc. R. Soc. A: Math. Phys. Eng. Sci.* **472**, 20160451 (2016).

7 Kou, J., *et al*. Optimal structure of tree-like branching networks for fluid flow. *Phys. A: Stat. Mech*, **393**, 527-534 (2014).

8 Brown, G. O. The history of the Darcy-Weisbach equation for pipe flow resistance. *Environmental and water resources history*, 34-43 (2002).

9 Moody, L. F. Friction factors for pipe flow. *Trans. Am. Soc. Mech. Eng.* **66**, 671-678 (1944).
