# Supplementary material for: Universal Murray’s law for optimised fluid transport in synthetic structures
Source: Nat Commun. 2024 May 7;15:3652. doi: 10.1038/s41467-024-47833-0 (PMC11076523; doi:10.1038/s41467-024-47833-0)
Supplement: Supplementary file 3 — Description of Additional Supplementary Files [file 41467_2024_47833_MOESM3_ESM.pdf]

Description of Additional Supplementary Files

Supplementary Software 1

Description: Python codes for i) layer spacing measurement ii) Orientation degree measurement iii) Pore diameter measurement
